# Postoperative arginine-enriched immune modulating nutrition: Long-term survival results from a randomised clinical trial in patients with oesophagogastric and pancreaticobiliary cancer

**DOI:** 10.1016/j.clnu.2021.09.040

**Published:** 2021-11

**Authors:** Alfred Adiamah, Katie E. Rollins, Audrey Kapeleris, Neil T. Welch, Syed Y. Iftikhar, Simon P. Allison, Dileep N. Lobo

**Affiliations:** a Nottingham Digestive Diseases Centre and National Institute for Health Research Nottingham Biomedical Research Centre, Nottingham University Hospitals NHS Trust and University of Nottingham, Nottingham, UK; b University Hospitals of Derby and Burton NHS Foundation Trust, Royal Derby Hospital, Uttoxeter Road, Derby, UK; c MRC Versus Arthritis Centre for Musculoskeletal Ageing Research, School of Life Sciences, University of Nottingham, Queen's Medical Centre, Nottingham, UK

**Keywords:** Immune modulating nutrition, Arginine, Oesophagogastric cancer, Pancreaticobiliary cancer, Long-term survival

## Abstract

**Background & aims:**

Immune modulating nutrition (IMN) has been shown to reduce postoperative infectious complications and length of stay in patients with gastrointestinal cancer. Two studies of IMN in patients undergoing surgery for head and neck cancer also suggested that this treatment might improve long-term survival and progression-free survival. In the present study, we analysed follow-up data from our previous randomised controlled trial of IMN, in patients undergoing surgery for oesophagogastric and pancreaticobiliary cancer, in order to evaluate the long-term impact on survival of postoperative IMN *versus* an isocaloric, isonitrogenous control feed.

**Methods:**

This study included patients undergoing surgery for cancers of the pancreas, oesophagus and stomach, who had been randomised in a double-blind manner to receive postoperative jejunostomy feeding with IMN (Stresson, Nutricia Ltd.) or an isonitrogenous, isocaloric feed (Nutrison High Protein, Nutricia) for 10–15 days. The primary outcome was long-term overall survival.

**Results:**

There was complete follow-up for all 108 patients, with 54 patients randomised to each group. There were no statistically significant differences between groups by demographics [(age, p = 0.63), sex (p = 0.49) or site of cancer (p = 0.25)]. 30-day mortality was 11.1% in both groups. Mortality in the intervention group was 13%, 31.5%, 70.4%, 85.2%, 88.9%, and 96.3% at 90 days, and 1, 5, 10, 15 and 20 years respectively. Corresponding mortality in the control group was 14.8%, 35.2%, 68.6%, 79.6%, 85.2% and 98.1% (p > 0.05 for all comparisons).

**Conclusion:**

Early postoperative feeding with arginine-enriched IMN had no impact on long-term survival in patients undergoing surgery for oesophagogastric and pancreaticobiliary cancer.

## 1 Introduction

The 5-year survival rate for oesophagogastric and pancreaticobiliary cancers is amongst the worst of all tumour types [1]. Surgical resection, which is the primary curative treatment, can suppress immune function and increase the risk of infection. Many patients with oesophagogastric and pancreaticobiliary cancer also suffer from malnutrition, which impairs immune function and leads to a higher incidence of postoperative complications, further impeding recovery.

Enteral feeding formulae containing key immune modulating nutrients such as l-arginine, l-glutamine, nucleotides, and ω-3 fatty acids have been designed to counteract these impairments in immunity as well as to counterbalance the catabolic response to surgery [2,3].

A meta-analysis of 35 randomised control trials (RCT) in patients, receiving preoperative l-arginine supplemented diets, found a 41% reduction in infectious complications and a 2-day reduction in hospital stay [2]. However, despite the apparent protective effect of perioperative immune modulating nutrition (IMN) many of these studies have failed to show any improvement in mortality [[2], [3], [4]]. Our own RCT from 2006 found no difference in 30-day mortality rate between patients receiving postoperative IMN and those given a standard isocaloric, isonitrogenous enteral feed [5]. However, two studies in patients undergoing surgery for head and neck cancers appear to show improved long-term survival in patients randomised to an arginine-containing feed arm when compared with controls [6,7].

The aim of this study was to analyse long-term survival from our previous RCT [5] to determine if arginine supplementation, as part of postoperative enteral IMN, in patients undergoing surgery for oesophagogastric and pancreaticobiliary cancer, improved long-term survival when compared with an isocaloric isonitrogenous control feed.

## 2 Methods

The detailed methodology of this study was published in our original paper [5] and may be summarised as follows:

### 2.1 Study design and setting

The original prospective, double-blind RCT [5] was set in Nottingham University Hospitals NHS Trust (Queen's Medical Centre and City Campuses) and the Royal Derby Hospital. It included adult patients undergoing elective resection for oesophagogastric and pancreaticobiliary cancer between January 2000 and June 2003. Patients were randomised, using a stratified minimisation design, to the experimental or control group, which were the same in terms of disease and body mass index (BMI) (<19 or ≥19 kg/m^2^). Patients with metastatic or unresectable disease, pregnant patients and those on immunosuppressive drugs were excluded.

### 2.2 Clinical management

All patients underwent surgical resection and needle catheter feeding tubes (Freka® FCJ tube, Fresenius Kabi Ltd., Hamburg, Germany) were inserted into the proximal jejunum at the time of the operation.

### 2.3 Intervention and study groups

Jejunostomy feeding was commenced 4 h after completion of the operation at a rate of 25 ml/h on day 0 (day of the operation), 50 ml/h on day 1, and 75 ml/h thereafter. Feeds were delivered by an infusion pump for 20 h/day with a 4 h rest period, for 10–15 days after surgery. Group A received the experimental IMN feed (Stresson – Nutricia Ltd., Zoetermeer, Netherlands) while Group B received an isonitrogenous, isocaloric control feed (Nutrison High Protein – Nutricia Ltd.) (Table 1).

**Table 1:** **Feed composition** (key nutrients per litre).

	Feed A (Stresson)	Feed B (Nutrison High Protein)
Energy	1250 kcal (5.2 MJ)	1250 kcal (5.2 MJ)
Total Protein	75 g	75 g
Arginine	8.9 g	3.0 g
Glutamine	13.0 g	7.5 g
Cysteine	0.7 g	0.2 g
Total Fat	41.7 g	48.6 g
Long chain triglycerides	24.5 g	29.2 g
Medium chain triglycerides	17.2 g	19.4 g
Eicosapentaenoic acid	0.79 g	–
Docosahexaenoic acid	0.3 g	–
ω6:ω3 fatty acid ratio	3.45:1	5:1
Carbohydrate	145 g (46% energy)	129 g (42% energy)
Na^+^	50 mmol	34 mmol
K^+^	58 mmol	44 mmol
Osmolalilty (mOsm/kg)	380	270

### 2.4 End-points

The primary end-point of this follow-up study was long-term survival up to 20 years postoperatively. Deaths were recorded from the time of surgery to 1 March 2021 for all patients.

### 2.5 Statistical analysis

All analysis were undertaken using Stata v16.1 (StataCorp, Stata Statistical Software: Release 16, College Station, Texas, USA). Demographic characteristics of the cohort were assessed with the Student t-test and χ^2^ test. Differences were considered significant at p < 0.05. Standard survival methods and Cox regression models were used to define the overall survival.

### 2.6 Ethics and consent

The ethics committees of the three hospitals involved approved the study design for the original study [5]. Informed written consent was obtained from each participant prior to enrolment. No additional permissions were required for evaluation of their overall survival.

## 3 Results

Of the 108 patients included, 54 received the IMN feed and 54 received the isocaloric, isonitrogeneous control feed (Table 1). There were no statistically significant differences in the demographic characteristics of the patients (age, gender, BMI), or site of primary tumour or hospital of intended surgery (Table 2). The median follow-up time was 1.88 years (IQR 0.78–6.07-years).

**Table 2:** Demographic and disease profile.

	Group A (Stresson) n = 54	Group B (Nutrison High Protein) n = 54	P value (Test)
**Age** [Mean (SE) years]	65.7 (1.4)	66.6 (1.4)	0.63 (Student t-test)
**Gender** (M:F)	40:14	43:11	0.49 (χ^2^ test)
**BMI** (<19 kg/m^2^:≥19 kg/m^2^)	4:50	5:49	1.0 (χ^2^ test)
**Site of cancer** (oesophagus:stomach:pancreas)	36:11:7	28:18:8	0.25 (χ^2^ test)
**Hospital** (A:B:C^a^)	23:19:12	19:21:14	0.73 (χ^2^ test)

a A = Nottingham University Hospitals, Queen's Medical Centre Campus, B= Nottingham University Hospitals, City Hospital Campus, C = Royal Derby Hospital.

### 3.1 Mortality

There were no statistically significant differences in mortality at any of the time periods analysed when the two groups were compared (Table 3).

**Table 3:** Cumulative mortality.

Mortality	IMN Group	Control Group	p value (χ^2^ test)
30-day	11.1%	11.1%	1.00
90-day	13.0%	14.8%	0.78
1-year	31.5%	35.2%	0.84
4-year	59.3%	61.1%	0.84
5-year	70.4%	68.5%	0.83
10-year	85.2%	79.6%	0.45
15-year	88.9%	85.2%	0.57
20-year	96.3%	98.1%	0.56

### 3.2 Kaplan–Meier survival curves

**Fig. 1:**
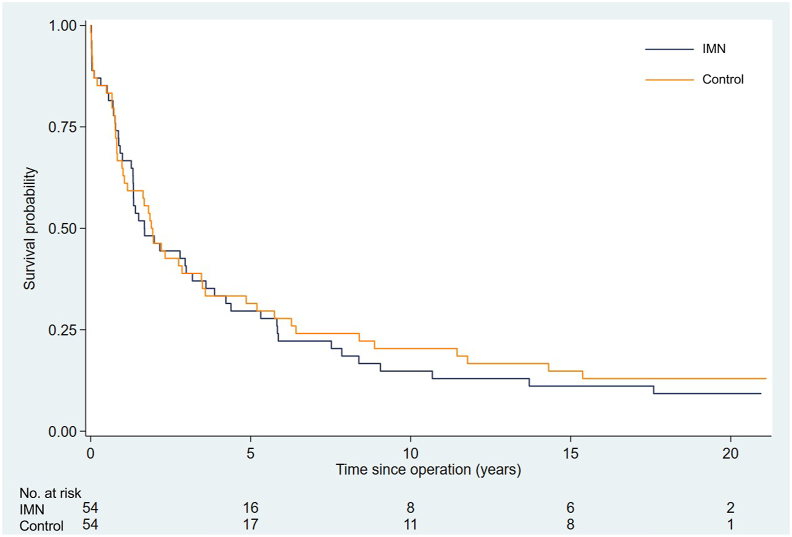
Kaplan–Meier curves showing cumulative postoperative survival in the immune modulating nutrition (IMN) and isocaloric, isonitrogenous (control) groups (p = 0.76).

Analysis of the survival curves with follow up to 20-years, demonstrated high early mortality with survival rates of less than 25% occurring by the 6th year in both groups. There was no statistically significant difference in long-term survival and by the 20th year of follow-up only one patient alive in the control group and two alive in the IMN group (Fig. 1). The hazards ratio (95% confidence intervals) for mortality was 1.066 (0.71–1.60), in the IMN group compared with controls. Using Cox regression analysis, demographic factors, disease characteristics, and hospital location did not influence the overall survival at any time point.

## 4 Discussion

This study found that early postoperative enteral feeding with arginine-enriched IMN conferred no additional benefit on either the short or long-term mortality when compared with an isocaloric, isonitrogeneous control formula in patients undergoing surgery for oesophagogastric and pancreaticobiliary cancer.

The benefits of IMN have been well described and include reductions in postoperative infectious complications and hospital length of stay [2,3,8]. The direct impact of IMN on both short-term and long-term mortality is less clear. Few RCTs in gastrointestinal surgery that investigate IMN were powered to identify a difference with mortality as the primary outcome. Nonetheless, several systematic reviews and meta-analysis in gastrointestinal surgery, have consistently shown no difference in mortality between the IMN and control groups [2,3,8]. One double-blind RCT in malnourished patients undergoing head and neck cancer surgery, who were given pre- and postoperative arginine enhanced IMN with a more than 10-year follow-up, found significantly improved long-term overall survival and long-term disease specific survival in patients who received an arginine enriched enteral formula compared with controls [6]. That study was, however, very small, with only 32 patients. At the time of that publication, all 15 patients in the control group and 14/17 patients in the arginine group had died. Also, although the original study [9] that preceded the long-term survival analysis [6] stated that there was no arginine in the feed received by the control group, that feed in fact contained casein which has between 1.9 and 4.7 g arginine/100 g casein [10]. A phase III multicentre RCT comparing an oral IMN with an isonitrogenous, isocaloric control supplement in 180 patients undergoing surgery and adjuvant chemo- and radiotherapy for head and neck cancer did not find any appreciable benefit on their primary end point, which was rate of grade 3 and 4 acute mucosal toxicity [7]. However, in a separate analysis of the subgroup of patients with a high (≥75%) compliance, both overall (81% *vs*. 61%) and progression-free survival (73% *vs*. 50%) were significantly greater in the experimental group than in the controls.

Our study, with a sample size of 108 demonstrated no significant difference in short- or long-term mortality. However, the overall volume of feed delivered was considerably lower than the target amount, but there was no statistically significant difference between the feed volumes delivered to each group [5]. Nevertheless, no meta-analysis has demonstrated a survival advantage with arginine-enriched IMN [2,3,8].

Our original study [5], on which this long-term analysis was based, was double-blind and stratified according to disease and BMI to ensure that operation type and degree of malnutrition were similar in the treatment and control groups and did not influence the outcome. However, there are some limitations that warrant further exploration. Firstly, the control feed was not completely inert as it contained some arginine albeit much less than the treatment feed (Table 1). It is uncertain if this amount of arginine would have been sufficient to affect the outcome. Additionally, whilst the IMN used in our study was Stresson, a study that reported survival benefit used Impact® (Nestlé, Vevey, Switzerland) which, in addition to arginine and ω-3 fatty acids has nucleotides but no glutamine [7]. Differences in the composition of the different enteral feeds available make it difficult to compare one with another. Finally, the timing of early enteral nutrition could also be important. A meta-analysis of 16 studies found that arginine-containing IMN (Impact®) given for 5–7 days before surgery resulted in less morbidity after elective surgery for gastrointestinal cancer, albeit not in improved short-term survival [8]. The total amount of IMN administered and the plasma concentration of immunonutrients achieved may also be important in terms of effectiveness. Because the tolerance to feeding is greater preoperatively than in the early postoperative period, the amount of feed that can be administered in trials of just postoperative feeding could possibly be less than required to have a significant effect. Therefore, the findings of this study, which relates to postoperative feeding cannot necessarily be extrapolated to IMN feeding in the preoperative setting, which in itself warrants a separate investigation.

Despite, and perhaps because of all these possible shortcomings of our study and those of others, there is currently very little evidence upon which to base a recommendation that, in patients undergoing major surgery for cancer, feeds containing extra immune modulating nutrients have any benefit in terms of mortality, either in the short-term or long-term, over standard feeds aimed at treating or preventing malnutrition and its consequences.

## Author contributions

Study Design: All.

Data collection: AA, KER, AK, SYI, DNL.

Data analysis: AA.

Data interpretation: All.

Writing of the manuscript: AA, KER, AK, DNL.

Critical review: All.

Final Approval All.

## Funding

This work was supported by the 10.13039/501100000265Medical Research Council [grant number MR/K00414X/1]; and 10.13039/501100000341Arthritis Research UK [grant number 19891]. The enteral feeds were provided free of cost by Nutricia Ltd. The views expressed are those of the authors and not necessarily those of the MRC, ARUK, NHS, the NIHR or the Department of Health. The funders of the study did not have any role in the study design, data collection, analysis and interpretation, writing of the paper or in the decision to submit the paper for publication.

## Conference presentation

This paper was presented to the ESPEN 2021 Virtual Congress and will be published in abstract form in Clinical Nutrition ESPEN.

## Conflict of interest

None of the authors has a direct conflict of interest to declare. The enteral feeds were provided free of cost by Nutricia Ltd.
